# *ACTN3* R577X polymorphism and long-term survival in patients with chronic heart failure

**DOI:** 10.1186/1471-2261-14-90

**Published:** 2014-07-24

**Authors:** Sabrina Bernardez-Pereira, Paulo Caleb Junior Lima Santos, Jose Eduardo Krieger, Alfredo Jose Mansur, Alexandre Costa Pereira

**Affiliations:** 1 Laboratory of Genetics and Molecular Cardiology, Heart Institute (InCor), University of São Paulo Medical School, Av. Dr. Enéas de Carvalho Aguiar, 44 Cerqueira César, São Paulo, SP, Brazil; 2 Fluminense Federal University, Rio de Janeiro, Brazil

**Keywords:** *ACTN3*, R577X, Polymorphism, Heart failure

## Abstract

**Background:**

Previous studies have shown the occurrence of actinin-3 deficiency in the presence of the R577X polymorphism in the *ACTN3* gene. Our hypothesis is that this deficiency, by interfering with the function of skeletal muscle fiber, can result in a worse prognosis in patients with chronic heart failure.

**Methods:**

A prospective cohort study was conducted from 2002 to 2004. The eligibility criteria included diagnosis of chronic heart failure stage C from different etiologies. We excluded all patients with concomitant disease that could be related to poor prognosis. *ACTN3* rs1815739 (R577X) polymorphism was detected by high resolution melting analysis. Survival curves were calculated with the Kaplan-Meier method and evaluated with the log-rank statistic. The relationship between the baseline variables and the composite end-point of all-cause death was assessed using a Cox proportional hazards survival model.

**Results:**

A total of 463 patients were included in this study. The frequency of the *ACTN3* 577X variant allele was 39.0%. The LVEF mean was 45.6 ± 18.7% and the most common etiology of this study was hypertensive. After a follow-up of five years, 239 (51.6%) patients met the pre-defined endpoint. Survival curves showed higher mortality in patients carrying RX or XX genotypes compared with patients carrying RR genotype (p = 0.01).

**Conclusion:**

R577X polymorphism in the *ACTN3* gene was independently associated with worse survival in patients with chronic heart failure. Further studies are necessary to ensure its use as a marker of prognosis for this syndrome.

## Background

Despite advances in the treatment of heart failure (HF) over the past few years, the understanding of disease mechanisms and prognosis still needs further investigation. The sequencing of the human genome and the resulting advances in molecular biology have opened new horizons since new prognostic biomarkers can serve as a good guide in the understanding and treatment of this complex disease [1].

It has been widely suggested that, although HF patients exhibit an abnormal cardiac output response to exercise, peripheral factors, mainly located in skeletal muscle, are the main determinants of low exercise tolerance in these patients and therefore involved in its prognosis [2]. However, despite the association between exercise capacity and prognosis of heart failure being well established, the mechanisms of this interaction remain to be better understood.

In skeletal muscle, the main protein components of the sarcomeric Z line are the alfa-actinins. In the heart and oxidative skeletal muscle fibers, the predominant expressed isoform is alfa-actinin-2 and in fast skeletal muscle fibers is alfa-actinin-3 (ACTN3) [3]. The *ACTN3* gene, located on 11q13.1, is expressed only in fast twitch fibers, which trigger faster and generate more force than alfa-actinin-2. The R577X polymorphism (rs1815739) has been identified in the *ACTN3* gene and it results in a complete loss of the actinin-3 protein in XX homozygotes and partial loss in heterozygote carriers [4].

In this scenario, the main aim of this study was to assess whether *ACTN3* R577X polymorphism is associated with mortality in heart failure patients.

## Methods

### Patient population

A prospective cohort study was conducted from 2002 to 2004 and included heart failure patients from Heart Institute of University of São Paulo Medical School, São Paulo, Brazil. The endpoint defined for this study was all-cause death. Follow-up by medical attempts or by telephone contact was performed each year until 2009. The study protocol was approved by the Ethics Committee from Hospital das Clinicas (CAPPesq) and all participants provided their written informed consent to participate in this study [5].

The eligibility criteria was described previously [5]. Patients were enrolled when the presence of symptomatic heart failure (stage C) was clinically diagnosed, functional class III-IV and age over 18 years. Different etiologies were included. Ischemic cardiomyopathy was defined when a clear history of previous myocardial infarction without other cause of left ventricular dysfunction or through coronary angiography. Ischemic cardiomyopathy was also excluded for all patients with diagnosis of idiopathic dilated cardiomyopathy through coronary angiography. Patients with prior myocardial infarction (<3 months), unstable angina, hypertrophic cardiomyopathy, valve heart disease candidates to surgical treatment, obstructive pulmonary disease, severe renal or hepatic dysfunction, current history of cancer, severe peripheral arterial disease, cerebrovascular disease and active infection were excluded.

### Genotyping

Genomic DNA from subjects was extracted from peripheral blood following standard salting-out procedure. Genotypes for the *ACTN3* rs1815739 (R577X) polymorphism were detected by polymerase chain reaction (PCR) followed by high resolution melting (HRM) analysis with the Rotor Gene 6000® instrument (Qiagen, Courtaboeuf, France) [6]. Amplification of the fragments was performed using the primer sense 5'-TCAGTTCAAGGCAACACTGC-3' and antisense 5'-CTTCTGGATCTCACCCTGGA-3'. A 35-cycle PCR with addition of fluorescent DNA-intercalating SYTO9® (1.5 μM; Invitrogen, Carlsbad, USA) was carried out with the following conditions: denaturation of the template DNA for first cycle of 94°C for 120 s, denaturation of 94°C for 20 s, annealing of 60°C for 20 s, and extension of 72°C for 20 s. In the HRM phase, Rotor Gene 6000® (Qiagen, Courtaboeuf, France) measured the fluorescence in each 0.1°C temperature increase in the range of 70-94°C. Melting curves were generated by the decrease in fluorescence with the increase in the temperature; nucleotide changes resulting from different curve patterns were analyzed and genotyped. Samples of the three observed curves were sequenced (ABI 3500XL Sequencer®, Applied Biosystems, Foster City, CA, USA) to confirm the genotypes indicated by HRM. The two methods gave identical results and, wild-type, heterozygous and homozygous genotypes for the *ACTN3* rs1815739 577X allele could be easily discernible by HRM analysis [7].

### Statistical analysis

Categorical variables were presented as percentage and continuous variables were presented as mean ± standard deviation. Demographic, biochemical, echocardiographic, and clinical data were analyzed using Student’s t test for continuous variables and the Chi-square test or Fisher’s test for categorical variables. Survival curves were constructed with the Kaplan–Meier method and differences between the curves were evaluated with the log-rank statistic. Cox proportional hazard model was performed to identify hazard ratios (HR) due to *ACTN3* polymorphism for the endpoint event, adjusted for age, gender, body mass index, ethnicity, left ventricular ejection fraction (LVEF), etiology (Chagasic was used as the reference category due to its worse prognostic), hemoglobin, and creatinine. These covariates were added in the model and were based on their clinical relevance regarding to heart failure. Our sample size provides 80% power to detect an association with the endpoint event with an effect size of 1.4 for the *ACTN3* R577X polymorphism. Hardy-Weinberg equilibrium analysis was estimated by the Chi-square test. All statistical analyses were carried out using SPSS software (version 16.0, IBM, New York, NY), with the level of significance set at p <0.05.

## Results

Of the 463 patients (mean age 58 ± 14), 283 (61.1%) were male and 345 (74.5%) were Whites. The LVEF mean was 46 ± 19% and the most frequent etiologies were hypertensive, ischemic, and valve diseases, with frequencies of 28.9%, 28.7% and 15.3%, respectively. After a follow-up of five years, 239 (51.6%) patients met the pre-defined study endpoint, the dropout was of 22.0% (n = 102), and the mean follow-up was of 1,152 days.

The frequency of the *ACTN3* 577X variant allele was 39.0% and the distribution of the genotypes was 15.6% (n = 72) for homozygous, 46.0% (n = 213) for heterozygous and 38.4% (n = 178) for wild-type genotype. The genotypic distribution for the *ACTN3* R577X polymorphism was in accordance with the Hardy–Weinberg equilibrium (X^2^ = 0.38, p = 0.53).

**Figure 1:**
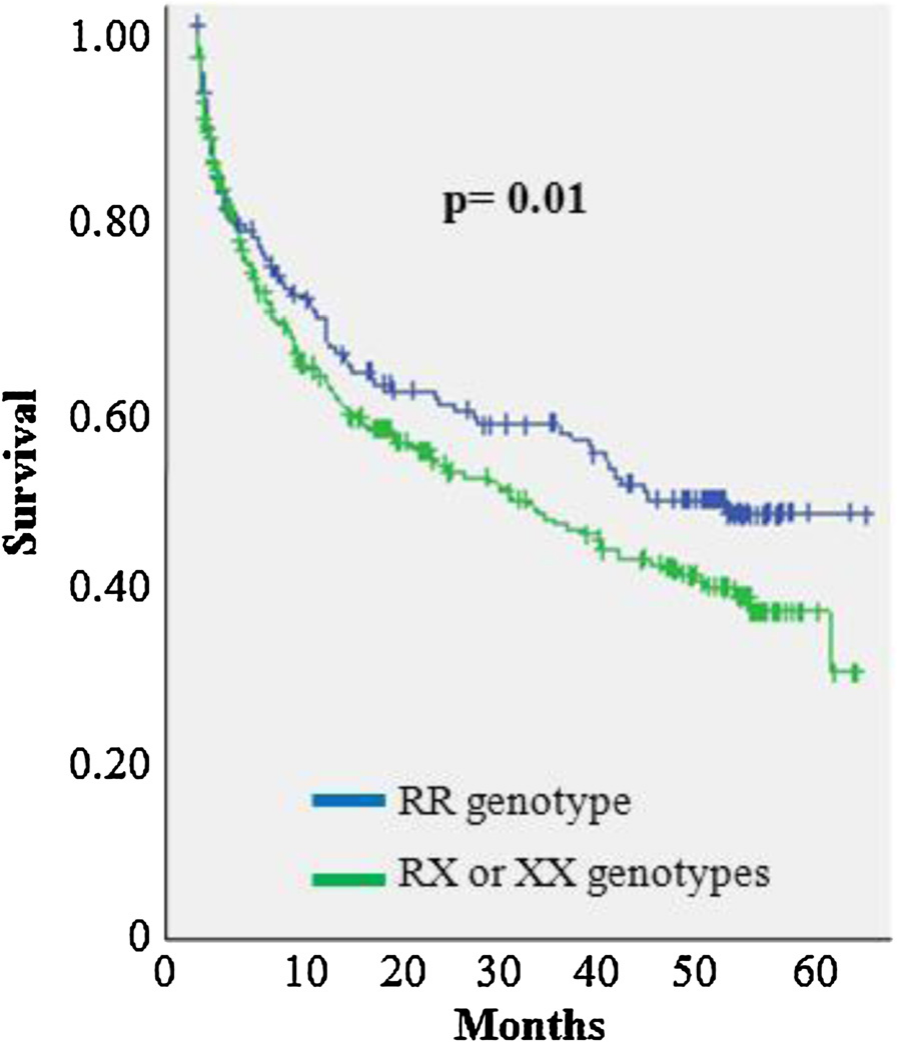
**Survival curves according to *ACTN3 *** **R577X polymorphism of the patients with heart failure.**

Table 1 shows baseline demographic, biochemical, echocardiographic, and clinical characteristics according to *ACTN3* R577X polymorphism of the patients with heart failure. These variables did not present significant difference according to genotypes. Figure 1 shows survival curves with higher mortality in patients carrying RX or XX genotypes compared with patients carrying RR genotype (p = 0.01). Furthermore, in a Cox proportional hazards survival model, the presence of the RX or XX genotypes in patients with heart failure was independently associated with higher hazard ratio compared with carriers of RR genotype (HR 1.72, 95% CI 1.14-2.62, p = 0.01) (Table 2), adjusted for covariates. Hemoglobin, creatinine, LVEF, and hypertensive etiology were also significant factors in the model.

**Table 1:** **Baseline demographic, biochemical, echocardiographic, and clinical characteristics according to *ACTN3 *R577X polymorphism of the patients with heart failure**

	**RR (n = 178)**	**RX + XX (n = 285)**	**p value**
**Gender (male), %**	64.6	58.9	0.22
**Age, years**	57 ± 14	58 ± 14	0.27
**Body mass index, kg/m**^**2**^	26 ± 5	25 ± 5	0.23
**Ethnicity (Whites), %**	68.5	78.2	0.09
**Heart failure etiology, %**			
**Chagasic**	9.6	11.9	
**Idiopathic**	9.6	10.5	
**Hypertensive**	35.4	24.9	0.29
**Ischemic**	25.3	30.9	
**Valve disease**	14.5	15.8	
**Other**	5.6	6.0	
**Hypertension, %**	62.4	62.8	0.92
**Diabetes, %**	25.3	25.6	0.93
**Current smoker, %**	12.4	7.0	0.06
**Total cholesterol, mg/dL**	193 ± 49	190 ± 52	0.57
**LDL-C, mg/dL**	123 ± 38	119 ± 44	0.34
**HDL-C, mg/dL**	46 ± 15	45 ± 15	0.69
**Triglycerides, mg/dL**	121 ± 68	123 ± 6.6	0.75
**Glycemia, mg/dL**	115 ± 38	109 ± 49	0.27
**Creatinine, mg/dL**	1.4 ± 0.8	1.4 ± 0.7	0.92
**Frequency of death, %**	46.6	54.7	0.08
**Systolic blood pressure, mmHg**	123 ± 33	121 ± 30	0.48
**Diastolic blood pressure, mmHg**	78 ± 20	75 ± 17	0.15
**Heart rate, ppm**	80 ± 14	79 ± 12	0.31
**LVEF, %**	46 ± 18	45 ± 19	0.71
**LVM, g**	259 ± 98	245 ± 83	0.12
**LVEDD, mm**	62 ± 12	62 ± 11	0.97
**LVESD, mm**	49 ± 15	49 ± 14	0.77

*LDL-C*: Low-density lipoprotein cholesterol; *HDL-C*: High-density lipoprotein cholesterol; *LVEF*: Left ventricular ejection fraction; *LVM*: Left ventricular mass; *LVEDD*: Left ventricular end-diastolic diameter; *LVESD*: Left ventricular end-systolic diameter.

**Table 2:** Cox proportional-hazards model for mortality in patients with heart failure

**Variables**	**HR**	**95% CI**	***p *** **value**
**RX or XX genotypes for the *ACTN3 *** **R577X**	1.72	1.14-2.62	0.01
**Gender (male)**	1.19	0.78-1.81	0.42
**Age** (years)	1.00	0.98-1.02	0.70
**Body mass index** (kg/m^2^)	0.99	0.95-1.04	0.93
**Ethnicity** (White vs Non-White)	1.24	0.78-1.94	0.36
**Hemoglobin** (g/dL)	0.87	0.79-0.94	0.002
**Creatinine** (mg/dL)	2.16	1.69-2.76	0.001
**LVEF** (%)	0.98	0.97-0.99	0.02
**Etiology**			
Chagasic (reference)	1.00		
Idiopathic	0.58	0.28-1.21	0.15
Hypertensive	0.50	0.26-0.97	0.04
Ischemic	0.74	0.42-1.32	0.31
Valve disease	0.70	0.35-1.40	0.32

*HR*: Hazard ratio; *LVEF*: Left ventricular ejection fraction.

## Discussion

Our findings suggest that heart failure patients carrying X allele of the *ACTN3* R577X polymorphism had significantly shorter survival time compared with patients with the RR genotype and the presence of the X allele conferred a worse prognosis. Known factors of worse prognosis in HF were also significant in our model as levels of creatinine, hemoglobin and LVEF.

In this study, hypertensive etiology conferred a better prognosis when compared with other causes of HF. However, other authors have already associated different etiologies with prognosis. Felker et al. showed a better prognosis in patients with peripartum cardiomyopathy while a worse prognosis has been demonstrated in patients with cardiomyopathy due to infiltrative myocardial diseases, HIV infection, or doxorubicin therapy [8]. Nevertheless Ochiai et al. have shown that Chagas disease predicted low cardiac output in decompensated severe heart failure and was associated with high levels of BNP and a worse prognosis, independently from lower ejection fraction [9]. But, these findings on HF prognosis scenario need further investigation.

This is the first study to investigate the possible association of the *ACTN3* gene with the prognosis of heart failure. Previous studies have associated this gene with human physical performance and elite athletic performance [10-13]. The functions of actinin-3 are likely to include a structural role in the maintenance of muscle mechanical integrity and other functions related to muscle signaling and metabolism and *ACTN3* R577X polymorphism leads to its deficiency [14]. Some authors showed the frequency of R wild-type allele was higher in sprint and power athletes who specifically require optimal fast fiber performance, while the X variant allele was more frequent in endurance athletes who rely predominantly on slow muscle fibers [15]. Additionally, a recent systematic review and meta-analysis brought a stronger evidence of the influence of the genetic profile with physical performance in athletes with positive association between *ACTN3* R allele and power events [16].

Furthermore R577X has been studied in the general population and it has been reported as one of many genetic factors that may influence muscle function [13]. Patients with chronic HF often report skeletal muscle weakness [17]; however, its mechanisms have not been clearly defined. Some authors consider that contractility is altered because of intracellular changes of Ca^2+^ metabolism [2], while Sullivan et al. demonstrated a decrease in slow twitch type I fibers, and a higher percentage of type IIb fast twitch fibers in chronic HF patients [18].

The recent HF ACTION trial demonstrated exercise to be associated with a significant reduction in all cause and cardiovascular mortality as well as hospital admissions [19]. The deficient muscle fiber type II in X allele carriers could justify low functional capacity and consequent worsening of survival.

The limitations of our study are the complex interacting factors that influence muscle performance. It is difficult to decide whether muscle function altered by genetic factors and training state could influence survival in chronic HF. Moreover we did not evaluate the physical capacity and muscular strength for more accurate testing in these patients. Thus, this study serves to generate hypotheses and requires further studies to confirm the possible interactions of gene-outcome in HF patients. In addition, we suggested that the R577X polymorphism might be used as a prognostic marker in heart failure. However, validation in a second population and analysis of analytical parameters of the biomarker are needed.

## Conclusion

Our study suggested that R577X polymorphism in the *ACTN3* gene modulates survival and could be used, in the future, as a prognostic marker in heart failure.

## Competing interests

The authors declare that they have no competing interests.

## Authors’ contributions

SB and PCJLS performed the statistical analysis and drafted the manuscript. AJM, ACP, and JEK conceived the study, and participated in its design. All authors read and approved the final manuscript.

## Pre-publication history

The pre-publication history for this paper can be accessed here:

http://www.biomedcentral.com/1471-2261/14/90/prepub
